# Estimating the Trial-by-Trial Learning Curve in Perceptual Learning with Hierarchical Bayesian Modeling

**DOI:** 10.21203/rs.3.rs-3649060/v1

**Published:** 2023-11-23

**Authors:** Yukai Zhao, Jiajuan Liu, Barbara Anne Dosher, Zhong-Lin Lu

**Affiliations:** Center for Neural Science, New York University, New York, USA; Department of Cognitive Sciences and Institute of Mathematical Behavioral Sciences, University of California, Irvine, CA, USA; Department of Cognitive Sciences and Institute of Mathematical Behavioral Sciences, University of California, Irvine, CA, USA; Division of Arts and Sciences, NYU Shanghai, Shanghai, China; Center for Neural Science and Department of Psychology, New York University, New York, USA; NYU-ECNU Institute of Brain and Cognitive Neuroscience, Shanghai, China

**Keywords:** Perceptual Learning, Learning curve, Hierarchical Bayesian modeling, Multi-component Processes

## Abstract

The learning curve serves as a crucial metric for assessing human performance in perceptual learning. It may encompass various component processes, including general learning, between-session forgetting or consolidation, and within-session rapid relearning and adaptation or deterioration. Typically, empirical learning curves are constructed by aggregating tens or hundreds of trials of data in blocks or sessions. Here, we devised three inference procedures for estimating the trial-by-trial learning curve based on the multi-component functional form identified in Zhao et al. (submitted): general learning, between-session forgetting, and within-session rapid relearning and adaptation. These procedures include a Bayesian inference procedure (BIP) estimating the posterior distribution of parameters for each learner independently, and two hierarchical Bayesian models (HBMv and HBMc) computing the joint posterior distribution of parameters and hyperparameters at the population, subject, and test levels. The HBMv and HBMc incorporate variance and covariance hyperparameters, respectively, between and within subjects. We applied these procedures to data from two studies investigating the interaction between feedback and training accuracy in Gabor orientation identification across about 2000 trials spanning six sessions (Liu et al., 2010, 2012) and estimated the trial-by-trial learning curves at both the subject and population levels. The HBMc generated best fits to the data and the smallest half width of 68.2% credible interval of the learning curves compared to the BIP and HBMv. The parametric HBMc with the multi-component functional form provides a general framework for trial-by-trial analysis of the component processes in perceptual learning and for predicting the learning curve in unmeasured time points.

## INTRODUCTION

The learning curve is a fundamental empirical measure of perceptual learning. It not only provides a depiction of the time course of learning but also assesses specificity and transfer of learning (see chapter 3 of Dosher & Lu, 2020). It has also been used to evaluate the functional form of learning (e.g., power or exponential function) (Cochrane & Green, 2021; Dosher & Lu, 2007) and, in some cases, contributions of multiple component processes, such as between-session improvements due to consolidation (Karni et al., 1994; McDevitt et al., 2014; Sasaki & Watanabe, 2015; Stickgold et al., 2002; Tamaki, Berard, et al., 2020; Tamaki et al., 2019; Tamaki, Wang, et al., 2020; Yotsumoto et al., 2009) or so-called off-line gain (Bang et al., 2018; Shibata et al., 2017), or alternatively between-session losses due to forgetting (Beard et al., 1995; Mascetti et al., 2013); and/or within-session performance reductions due to processes of adaptation (Censor et al., 2006, 2016; Sagi, 2011a) or general deterioration associated with fatigue or inattention (Dosher & Lu, 2020; Zenger-Landolt & Fahle, 2001).

In most perceptual learning studies, the empirical learning curve is constructed by aggregating data in blocks or sessions, often based on tens or hundreds of trials (Green et al., 2018; Lu & Dosher, 2022; Sagi, 2011b; Dosher & Lu, 2020). While the aggregated data consist of average performance accuracy or response times in the constant stimuli paradigm (Ball & Sekuler, 1982; Fahle et al., 1995; Fahle & Morgan, 1996; Karni & Sagi, 1991; Kattner, Cochrane, & Green, 2017a; Petrov et al., 2005, 2011), average contrast threshold or the difference threshold between the to-be-discriminated stimuli is often used in adaptive training paradigms (Donovan et al., 2015; Dosher & Lu, 1998; Polat et al., 2004; Xiao et al., 2008). However, assessing the empirical learning curve with aggregated data may obscure important characteristics of the process (Kattner, Cochrane, & Green, 2017b; Yang et al., 2022; Zhao et al., Submitted; Cochrane, Green & Lu, Submitted).

Several research groups have developed parametric methods to estimate the empirical learning curve on a trial-by-trial basis (Kattner, Cochrane, & Green, 2017a; Zhang et al., 2019a; Cochrane, Green & Lu, Submitted). By modeling the thresholds and bias of the psychometric function as specific parametric functions of time or trial number during perceptual learning, researchers were able to construct high-quality trial-by-trial learning curves for each observer during training and transfer, based on the model’s best-fitting parameters (Dale et al., 2021; Kattner, Cochrane, & Green, 2017a; Zhang et al., 2019a, 2019b).

One prerequisite of the parametric methods is a suitable parametric functional form. In this study, we developed and applied inference procedures to estimate the trial-by-trial learning curve in a Gabor orientation identification task based on the multi-component functional form (MCFF) of perceptual learning revealed in Zhao et al (submitted) for this task.

**Figure 1.**
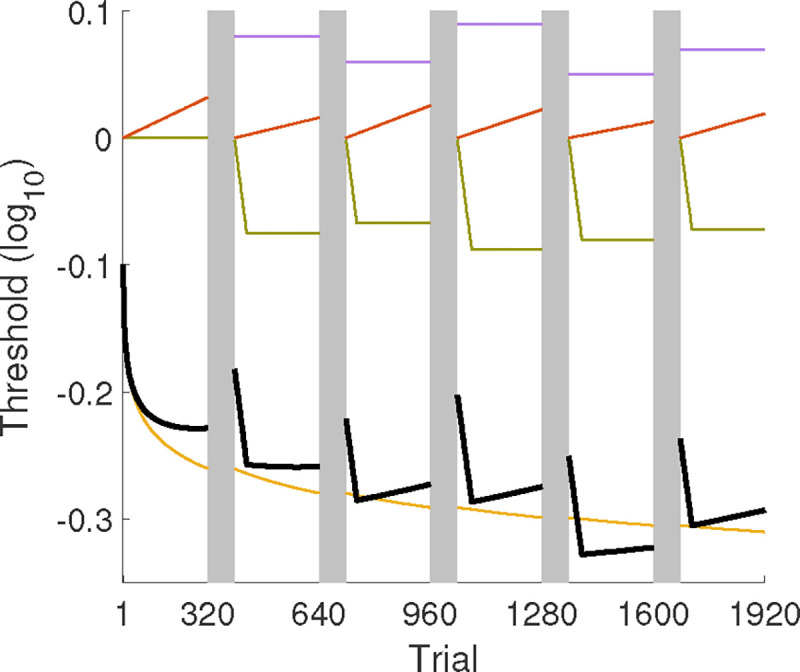
The trial-by-trial generative model of the learning curve (black curve) across six sessions, comprising four latent component processes: general learning (yellow), between-session forgetting (purple), within-session re-learning (olive), and within-session adaptation (orange).

Zhao et al. (submitted) developed and applied three non-parametric inference procedures to the data from two studies that investigated the interaction between feedback and training accuracy in Gabor orientation identification over approximately 2000 trials across six sessions and estimated the learning curve with block sizes of 20,40,80,160, and 320 trials. Analysis at the scale of 20 trials per block identified significant contributions from general learning, between-session forgetting, and rapid relearning and adaptation within sessions, resulting in an MCFF with four latent component processes: general learning, between-session forgetting, within-session re-learning, and within-session adaptation. An underlying learning curve (black curve) from the MCFF across six sessions is depicted in Figure 1, with the four component processes depicted in yellow, purple, olive, and orange curves.

In the present study, we developed three parametric inference procedures to fit the MCFF to the trial-by-trial perceptual learning data in Liu et al. (2010, 2012):

1. Bayesian Inference Procedure (BIP): This method estimates the posterior distribution of the parameters of the MCFF for each subject independently.
2. Hierarchical Bayesian Model with Population, Subject, and Test Levels, with Variance but no Covariance Hyperparameters at the Population level (HBMv): This model estimates the joint posterior MCFF hyperparameter and parameter distribution across all subjects, without considering the covariance within and between subjects.
3. Hierarchical Bayesian Model with Population, Subject, and Test Levels, Incorporating Covariance Hyperparameters at the Population Level (HBMc): This model estimates the joint posterior MCFF hyperparameter and parameter distribution across all subjects, considering the covariance within and between subjects.

The HBMs consist of three levels of the hierarchy: population, subject, and test, in which all subjects belong to a population and may in principle run the same experiment (called “test”) multiple times. In the HBMv and HBMc, the distributions of MCFF parameters at the test level are conditioned on the hyperparameter distributions at the subject level, which, in turn, are conditioned on the hyperparameter distribution at the population level. The HBMc includes covariance hyperparameters at the population and subject levels to capture relationships between and within subjects, while the BIP and HBMv do not.

Most perceptual learning experiments use a hierarchical experimental design structure (Kim et al., 2014; Yin et al., 2018), in which the study population is divided into multiple groups with different training protocols, each consisting of multiple subjects. Hierarchical models allow us to effectively combine information across subjects and groups while preserving heterogeneity (Kruschke, 2014; Rouder & Lu, 2005). The HBM typically consists of sub-models and probability distributions at multiple levels of the hierarchy and can compute the joint posterior distributions of the parameters and hyperparameters using Bayes’ theorem based on all available data (Kruschke, 2014; Kruschke & Liddell, 2018). Taking advantage of conditional dependencies within and across levels, the HBM often reduces the variance of the estimated posterior distributions by decomposing variabilities from different sources using parameters and hyperparameters (Song et al., 2020) and shrinking estimated parameters at lower levels towards the modes of higher levels when there is insufficient data at the lower level (Kruschke, 2014; Rouder et al., 2003; Rouder & Lu, 2005). It has found applications in many perception and cognitive science studies (Ahn et al., 2011; Lee, 2006; Merkle et al., 2011; Palestro et al., 2018; Prins, 2019; Rouder et al., 2003; Rouder & Lu, 2005; Wilson et al., 2020; Zhao et al., 2023c, 2023b, 2023a; Zhao, Lesmes, Dorr, et al., 2021; Zhao, Lesmes, Hou, et al., 2021)..

In this paper, we introduce the multi-component functional form (MCFF) along with the contrast threshold psychometric function as the generative model of trial-by-trial performance in perceptual learning. We provide an overview of the Bayesian inference procedure (BIP), which performs an independent estimation of the posterior distribution of MCFF parameters and, therefore trial-by-trial learning curve for each subject. Subsequently, we present HBMv and HBMc models which are designed to collectively estimate the joint posterior distribution of the MCFF hyperparameters and parameters and, therefore the trial-by-trial learning curves for all subjects. We applied these procedures to data from two studies that investigated the interaction between feedback and training accuracy in Gabor orientation identification over approximately 2000 trials across six sessions (Liu et al., 2010, 2012). Our analysis involved comparing the goodness of fit and variability of estimated trial-by-trial learning curves obtained from the three methods. Furthermore, we assessed the quality of the predicted learning curves from the HBMc for subjects with no, one session, and two sessions of training data.

## METHODS

### Data

We obtained data from two previously published studies (Liu et al., 2010, 2012) that evaluated the interaction between feedback and training accuracy in a Gabor orientation identification task, where the orientations were $45\pm 10^{\circ }$. The experiments used adaptive staircases to control the performance accuracy over the course of training. The first dataset was comprised of four groups: low training accuracy (65% correct) with and without feedback, and high training accuracy (85% correct) with and without feedback. The second dataset included six groups: low training accuracy (65% correct) with and without feedback, high training accuracy (85% correct) with and without feedback, and mixed training accuracy (65% and 85% correct) with and without feedback. Each experimental group included six naïve subjects.

We constructed six groups (g=1,2,…G;G=6) by combining subjects from the low (Group 1: without feedback; Group 2: with feedback) and high training accuracy (Group 5: without feedback; Group 6: with feedback) conditions across the two datasets. Subjects in the mixed training accuracy conditions without and with feedback were labeled as Groups 3 and 4 .

All participants in the published studies had normal or corrected-to-normal vision. They completed the orientation identification task using an accelerated stochastic staircase procedure (Kesten, 1958) with 320 trials in each of the six daily sessions (s=1,2,..S;S=6). Fifty-nine of them received 60–80 trials pre-training using a QUEST procedure (Watson & Pelli, 1983) in the beginning of the first session, while one subject did not receive pre-training. Detailed descriptions of the experimental procedures can be found in the original papers (Liu et al., 2010, 2012).

### Apparatus

The original experiments were conducted using MATLAB (MathWorks Corp., Natick, MA, USA) on a Macintosh Power PC G4 computer with a Nanao Technology Flexscan 6600 monitor. Subjects viewed the displays binocularly at 72 cm in a dimly lit room, and they used a chin rest to maintain their head positions. Data analyses were performed on a Dell computer with an Intel Xeon W-2145 @ 3.70GHz CPU (8 cores and 16 threads) and 64GB of installed memory (RAM). The analyses were conducted using MATLAB and JAGS (Plummer, 2003) in R (R Core Team, 2003).

## THEORETICAL FRAMEWORK

In this section, we first introduce the MCFF and the likelihood function. We then describe the Bayesian Inference Procedure (BIP), Hierarchical Bayesian Model with population, subject, and test levels with variance hyperparameters (HBMv), and Hierarchical Bayesian Model with population, subject, and test levels with covariance hyperparameters (HBMc).

To begin, each subject i∈[1,I] (where I=60) in each test j completed $T_{ij}$ trials in the study. We retain the index j for generality, although in this case, we set j=1 because all the subjects only ran the experiment once. The three inference methods were applied to the experimental data in a condition-blind manner, without considering the training accuracy and feedback conditions. Following group-blinded estimation, the data will be unblinded and the resulting characteristics of learning will be analyzed.

### Generative Model of Trial-by-trial Performance

#### The MCFF

The ${log}_{10}$ threshold learning curve is constructed by adding the contributions of all latent component processes in the MCFF (Figure 1). We consider four component processes:

1. General learning: $b-\gamma {log}_{10}(t)$, where t is the trial number, b represents the initial threshold and γ is the learning rate;
2. Between-session forgetting: This is depicted as a step function at the beginning of each daily session, characterized by a height $\delta_{s}$, which can vary across sessions (s);
3. Within-session rapid relearning: Modeled as an elbow function with a rapid linear learning rate $-\tau_{s}$ and an asymptotic level $-d_{s}$, both of which can vary across sessions (s);
4. Within-session adaptation or deterioration: Represented as a linear increasing function with a rate $\varphi_{s}$, which can vary across sessions (s).

In this study, we tested three versions of the MCFF. In the most saturated version considered in this study based the best model revealed in Zhao et al. (submitted), with K=14 parameters, both between-session forgetting $\left(\delta_{s}\right)$ and the asymptotic level of rapid relearning $d_{s}$ were free to vary across sessions. In one reduced model with K=6 parameters, we constrained $\delta_{s}$ and $d_{s}$ to be the same across sessions. In the most simplified model with K=2 parameters, we retained only general relearning and removed all other component processes. For clarity, Table 1 lists the correspondence between $\theta_{ijk}$ and the original MCFF parameters for the three versions of the MCFF.

**Figure 2.**
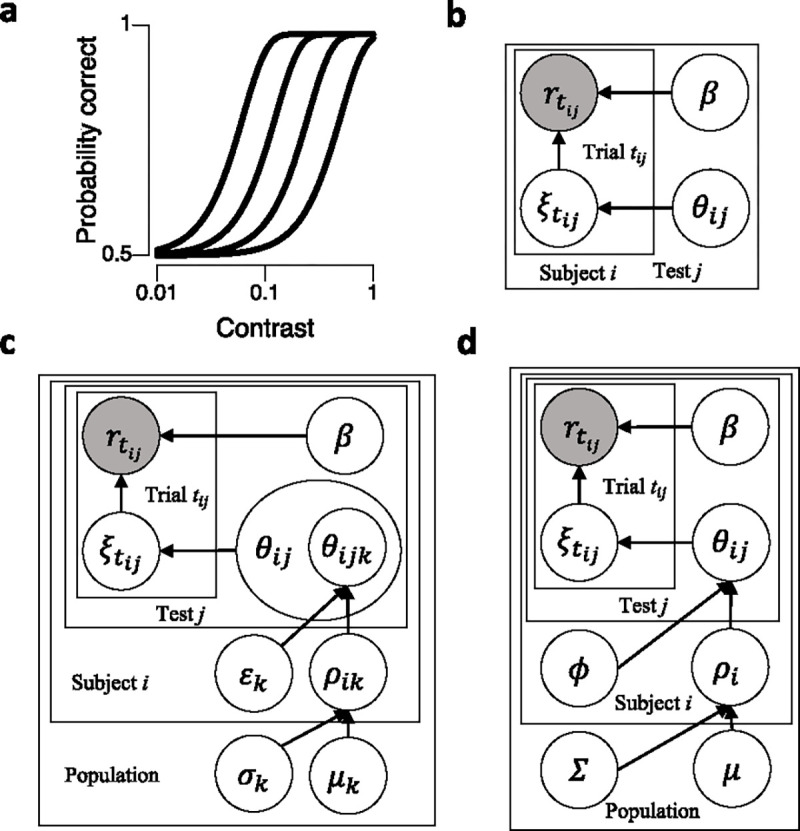
(a) Psychometric function: A family of psychometric functions parameterized with ${log}_{10}$ contrast thresholds $\xi_{t_{ij}}$ and slope β. They serve as the likelihood functions in the inference procedures. (b) Bayesian inference procedure (BIP): The BIP is used to compute the posterior MCFF parameter distributions for each subject independently. $\theta_{ij}$ represents MCFF parameters for subject i in test j. (c) Hierarchical Bayesian Model with variance hyperparameters (HBMv): The model calculates the joint distribution of MCFF parameters and hyperparameters across tests and subjects. It incorporates mean $\mu_{k}$ and standard deviation $\sigma_{k}$ hyperparameters for the population and mean $\rho_{ik}$ and standard deviation $\varepsilon_{k}$ hyperparameters for individual subjects. Notably, $\varepsilon_{k}$ are assumed to be the same for all subjects, where k is a single dimension of the hyperparameters. (d) Hierarchical Bayesian Model with covariance hyperparameters (HBMc). The HBMc estimates the joint distribution of MCFF parameters and hyperparameters across tests and subjects. It utilizes mean μ and covariance Σ hyperparameters for the population and mean $\rho_{i}$ and covariance ϕ hyperparameters for individual subjects. ϕ is assumed to be the same for all subjects.

On each trial $t_{ij}$, the ${log}_{10}$ contrast threshold $\xi_{t_{ij}}$ for subject i in test j can be calculated by adding the contributions of all the latent component processes with parameters $\theta_{ij}=\left(\theta_{ij1},\theta_{ij2},\ldots ,\theta_{ijK}\right)$. The probability of obtaining a correct response, denoted as $r_{t_{ij}}=1$, to a stimulus with contrast $c_{t_{ij}}$ in trial $t_{ij}$ is described using a Weibull psychometric function (Figure 2a):

(1a) $$p\left(r_{t_{ij}}=1|\theta_{ij},\beta ,c_{t_{ij}}\right)=g\lambda +(1-g)(g+(1-g)(1-exp\left(-{\left(\frac{c_{t_{ij}}}{\vartheta_{t_{ij}}}\right)}^{\beta }\right))),$$

(1b) $$\begin{array}{c} {log}_{10}\left(\vartheta_{t_{ij}}\right)=\xi_{t_{ij}}-\frac{1}{\beta }{log}_{10}\left(log(\frac{1-g}{1-p_{1.5}})\right). \end{array}$$

Here, in equation 1a,

- g=0.5 represents the guessing rate.
- β represents the slope of the Weibull psychometric function in a two-alternative forced-choice task (2AFC).
- λ=0.04 represents the lapse rate.
- $p_{1.5}=0.856$ is the probability of making a correct response when $d^{\prime }=1.5$ in a 2AFC task.

The probability of obtaining an incorrect response is given by:

(2) $$p\left(r_{t_{ij}}=0|\theta_{ij},\beta ,c_{t_{ij}}\right)=1-p\left(r_{t_{ij}}=1|\theta_{ij},\beta ,c_{t_{ij}}\right).$$

Equations 1 and 2 define the likelihood function by quantifying the probability of a correct or incorrect response based on stimulus contrast $c_{t_{ij}}$, the slope of the psychometric function β, and parameters of the $MCFF\theta_{ij}$. The overall probability of observing all the responses $\left(r_{1:T_{ij}}\right)$ for subject i in test j is determined by the product of individual probabilities $\left(r_{t_{ij}}|\theta_{ij},\beta ,c_{t_{ij}}\right)$ for all trials in that specific test:

(3) $$p\left(r_{1:T_{ij}}|\theta_{ij},\beta ,c_{1:T_{ij}}\right)=\prod_{1}^{T}\;p\left(r_{t_{ij}}|\theta_{ij},\beta ,c_{t_{ij}}\right).$$

### Bayesian Inference Procedure (BIP)

The Bayesian Inference Procedure (BIP) is used to estimate the posterior distribution of $\theta_{ij}$ from the trial-by-trial data $Y_{ij}=\{\left(r_{1:T_{ij}},c_{1:T_{ij}}\right)\}$ of subject i in test j via Bayes’ rule (Figure 2b):

(4) $$p\left(\theta_{ij},\beta |Y_{ij}\right)=\frac{\Pi_{1}^{T_{ij}}p\left(r_{t_{ij}}|\theta_{ij},\;\beta ,\;c_{t_{ij}}\right)p_{0}\left(\theta_{ij},\beta \right)}{\int \prod_{1}^{T_{ij}}\;\;p\left(r_{t_{ij}}|\theta_{ij},\;\beta ,{\;c}_{t_{ij}}\right)p_{0}\left(\theta_{ij},\beta \right)d\theta_{ij}d\beta }.$$

Here, $p\left(\theta_{ij},\beta |Y_{ij}\right)$ is the posterior distribution of $\theta_{ij}$ and β, which represents MCFF parameters and the slope of the psychometric functions, given the trial-by-trial data $Y_{ij}$, $p\left(r_{t_{ij}}|\theta_{ij},\beta ,c_{t_{ij}}\right)$ is the likelihood term, which quantifies the probability of observing the responses $r_{t_{ij}}$ given $\theta_{ij}$ and $c_{t_{ij}}$, $p_{0}\left(\theta_{ij},\beta \right)$ is the prior probability distribution of $\theta_{ij}$ and β. In this application, the prior of $\theta_{ij}$ is set as uniform distributions for each dimension k:

(5) $$p_{0}\left(\theta_{ijk}\right)=𝒰\left(\theta_{0k,min},\theta_{0k,max}\right),$$

where $\theta_{0k,min}$ and $\theta_{0k,max}$ are the lower and upper bounds of dimension k (Table 2). The denominator of equation (4) is an integral across all possible values of $\theta_{ij}$.

In the BIP, β is set to 2 for all subjects based on the results from the HBMv and HBMc to allow more stable estimates $\theta_{ij}$, because it is difficult to estimate based on data from a single subject with limited range of performance levels, but the MCFF parameters are estimated independently for each subject.

### Hierarchical Bayesian Model with Variance (HBMv)

The Hierarchical Bayesian Model with Variance (HBMv) is a three-level hierarchical Bayesian model used to estimate the joint posterior MCFF hyperparameter and parameter distribution across all subjects, without considering covariance within and between subjects (Figure 2c). This model includes probability distributions at three levels: population level, subject level, and test level.

#### Population Level:

The probability distribution of $\eta_{k}$, the $k^{th}$ dimension of the MCFF hyperparameter η at the population level, is modeled as a mixture of Gaussian distributions 𝒩 with mean $\mu_{k}$ and standard deviation $\sigma_{k}$, which in turn have distributions $p\left(\mu_{k}\right)$ and $p\left(\sigma_{k}\right)$:

(6a) $$p\left(\eta_{k}\right)=𝒩\left(\eta_{k},\mu_{k},\sigma_{k}\right)p\left(\mu_{k}\right)p\left(\sigma_{k}\right).$$

The probability distribution of η, p(η), is the product of the probability distributions across all dimensions of η:

(6b) $$p\left(\eta \right)=\prod_{k}\;𝒩\left(\eta_{k},\mu_{k},\sigma_{k}\right)p\left(\mu_{k}\right)p\left(\sigma_{k}\right).$$

#### Subject Level:

The probability distribution of $\tau_{ik}$, the $k^{th}$ dimension of hyperparameter $\tau_{i}$ of subject i at the subject level is modeled as a mixture of Gaussian distributions with mean $\rho_{ik}$ and standard deviation $\varepsilon_{k}$, with distributions $p\left(\rho_{ik}|\eta_{k}\right)$ and $p\left(\varepsilon_{k},\right)$:

(7a) $$p\left(\tau_{ik}|\eta_{k}\right)=𝒩\left(\tau_{ik},\rho_{ik},\varepsilon_{k}\right)p\left(\rho_{ik}|\eta_{k}\right)p\left(\varepsilon_{k}\right),$$

in which $\rho_{ik}$ is conditioned on $\eta_{k}$. The conditional probability distribution of $\tau_{i}$, $p\left(\tau_{i}|\eta \right)$, is the product of the conditional probability distributions across all dimensions of $\tau_{i}$:

(7b) $$p\left(\tau_{i}|\eta \right)=\prod_{k}\;𝒩\left(\tau_{ik},\rho_{ik},\varepsilon_{k}\right)p\left(\rho_{ik}|\eta_{k}\right)p\left(\varepsilon_{k}\right).$$

#### Test Level:

The probability distribution of parameters $\theta_{ijk}$ is conditioned on $\tau_{ik}$, $p\left(\theta_{ijk}|\tau_{ik}\right)$. The conditional probability distribution of $\theta_{ij},p\left(\theta_{ij}|\tau_{i}\right)$, is the product of the conditional probability distributions across all dimensions of $\theta_{ij}$:

(8) $$p\left(\theta_{ij}|\tau_{i}\right)=\prod_{k}\;p\left(\theta_{ijk}|\tau_{ik}\right).$$

The probability of obtaining all the data is computed using probability multiplication, which involves all levels of the model and the likelihood function based on the trial data:

(9) $$\begin{array}{c} p\left(Y_{1:I,1:J}|X\right)=\prod_{i=1}^{I}\;\prod_{j=1}^{J}\;\prod_{t_{ij}=1}^{T_{ij}}\;p\left(r_{1:T_{ij}}|\theta_{ij},\beta ,c_{1:T_{ij}}\right)p\left(\theta_{ij}|\tau_{i}\right)p\left(\tau_{i}|\eta \right)p(\eta )p(\beta )\\ =\prod_{i=1}^{I}\;\prod_{j=1}^{J}\;\prod_{t_{ij}=1}^{T_{ij}}\;p\left(r_{1:T_{ij}}|\theta_{ij},\beta ,c_{1:T_{ij}}\right)p(\beta )\prod_{k}^{K}\;p\left(\theta_{ijk}|\tau_{ik}\right)𝒩\left(\tau_{ik},\rho_{ik},\varepsilon_{k}\right)p\left(\rho_{ik}|\eta_{k}\right)p\left(\varepsilon_{k}\right)𝒩\left(\eta_{k},\mu_{k},\sigma_{k}\right)p\left(\mu_{k}\right)p\left(\sigma_{k}\right), \end{array}$$

where K=2,6, and 14 for the three versions of the MCFF, and $X=\left(\theta_{1:I,1:J,},\rho_{1:I,K},\mu_{K},\sigma_{K},\varepsilon_{K},\beta \right)$ are all the MCFF parameters and hyperparameters in the HBMv.

Bayes’ rule is used to compute the joint posterior distribution X, which includes all MCFF parameters and hyperparameters (Kruschke, 2014; Lee, 2011):

(10) $$p\left(X|Y_{1:I,1:J}\right)=\frac{\prod_{i=1}^{I}\;\;\Pi_{j=1}^{J}\Pi_{t_{ij=1}}^{T_{ij}}p\left(r_{1:T_{ij}}|\theta_{ij},\;\beta ,\;c_{1:T_{ij}}\right)p_{0}(\beta )\prod_{k}^{K}\;\;p\left(\theta_{ijk}|\tau_{ik}\right)𝒩\left(\tau_{ik},\rho_{ik},\varepsilon_{k}\right)p\left(\rho_{ik}|\eta_{k}\right)p_{0}\left(\varepsilon_{k}\right)𝒩\left(\eta_{k},\mu_{k},\sigma_{k}\right)p_{0}\left(\mu_{k}\right)p_{0}\left(\sigma_{k}\right)}{\int \prod_{i=1}^{I}\;\;\Pi_{j=1}^{J}\Pi_{T_{ij}=1}^{T_{ij}}p\left(r_{1:T_{ij}}|\theta_{ij},\;\beta ,\;c_{1:T_{ij}}\right)p_{0}(\beta )\prod_{k}^{K}\;\;p\left(\theta_{ijk}|\tau_{ik}\right)𝒩\left(\tau_{ik},\rho_{ik},\varepsilon_{k}\right)p\left(\rho_{ik}|\eta_{k}\right)p_{0}\left(\varepsilon_{k}\right)𝒩\left(\eta_{k},\mu_{k},\sigma_{k}\right)p_{0}\left(\mu_{k}\right)p_{0}\left(\sigma_{k}\right)dX},$$

where the denominator is an integral across all possible values of X and is a constant for a given dataset and $HBMv;p_{0}\left(\mu_{k}\right)$, $p_{0}\left(\sigma_{k}\right)$, $p_{0}\left(\varepsilon_{k}\right)$, and $p_{0}(\beta )$ are the prior distributions of $\mu_{k}$, σ, ε, and β:

(11a) $$p_{0}\left(\mu_{k}\right)=𝒰\left(\theta_{0k,min},\theta_{0k,max}\right),$$

(11b) $$p_{0}\left(\frac{1}{\sigma_{k}^{2}}\right)=\Gamma (15,1),$$

(11c) $$p_{0}\left(\frac{1}{\varepsilon_{k}^{2}}\right)=\Gamma (20,1),$$

(11d) $$p_{0}(\beta )=𝒰(1,4),$$

where $\theta_{0k,min}$ and $\theta_{0k,max}$ are defined in Table 2; Γ(λ,ϱ) is a Gamma distribution with shape parameter λ and rate parameter ϱ.

The HBMv estimates the joint posterior distributions of MCFF hyperparameters and parameters of all tests and subjects without considering covariance within and between subjects, while sharing a common slope parameter β across all tests and subjects.

### Hierarchical Bayesian Model with Covariance (HBMc)

The Hierarchical Bayesian Model with Covariance (HBMc) is a three-level hierarchical Bayesian model used to estimate the joint posterior MCFF hyperparameter and parameter distribution across all subjects, considering the covariance within and between subjects (Figure 2d). The HBMc includes probability distributions at three levels: population level, subject level, and test level.

#### Population Level:

The probability distribution of the MCFF hyperparameter η, which consists of all MCFF component parameters at the population level, is modeled as a mixture of K-dimensional Gaussian distributions with mean μ and covariance Σ, which have distributions of p(μ) and p(Σ):

(12) p(η)=𝒩(η,μ,Σ)p(μ)p(Σ).

#### Subject Level:

The probability distribution of the contrast threshold hyperparameter $\tau_{i}$ of subject i at the subject level is modeled as a mixture of K-dimensional Gaussian distributions with mean $\rho_{i}$ and covariance ϕ, with distributions $p\left(\rho_{i}|\eta \right)$ and p(ϕ):

(13) $$p\left(\tau_{i}|\eta \right)=𝒩\left(\tau_{i},\rho_{i},\phi \right)p\left(\rho_{i}|\eta \right)p(\phi ),$$

in which $\rho_{i}$ is conditioned on η.

#### Test Level:

$p\left(\theta_{ij}|\tau_{i}\right)$, the probability distribution of the parameters $\theta_{ij}$ is conditioned on $\tau_{i}$. The probability of obtaining the entire dataset is computed using probability multiplication, which involves all levels of the model and the likelihood function based on the trial data:

(14) $$p\left(Y_{1:I,1:J}|X\right)=\prod_{i=1}^{I}\;\;\prod_{j=1}^{J}\;\;\prod_{t_{ij}=1}^{T_{ij}}\;\;p\left(r_{1:T_{ij}}|\theta_{ij},\beta ,c_{1:T_{ij}}\right)p\left(\theta_{ij}|\tau_{i}\right)p\left(\tau_{i}|\eta \right)p(\eta )p(\beta )\;\;=\prod_{i=1}^{I}\;\;\prod_{j=1}^{J}\;\;\prod_{t_{ij}=1}^{T_{ij}}\;\;p\left(r_{1:T_{ij}}|\theta_{ij},\beta ,c_{1:T_{ij}}\right)p\left(\theta_{ij}|\tau_{i}\right)𝒩\left(\tau_{i},\rho_{i},\phi \right)p\left(\rho_{i}|\eta \right)p(\phi )𝒩(\eta ,\mu ,\Sigma )p(\mu )p(\Sigma )p(\beta ),$$

where $X=\left(\theta_{1:I,1:J},\rho_{1:I},\mu ,\phi ,\Sigma ,\beta \right)$ are all the parameters and hyperparameters in the HBMc.

Bayes’ rule is used to compute the joint posterior distribution of X, which includes all MCFF parameters and hyperparameters. This computation involves integrating over all possible values of X:

(15) $$p\left(X|Y_{1:I,1:J}\right)=\frac{\prod_{i=1}^{I}\;\Pi_{j=1}^{J}\Pi_{t_{ij=1}}^{T_{ij}}p\left(r_{1:T_{ij}}|\theta_{ij},\;\beta ,\;c_{1:T_{ij}}\right)p\left(\theta_{ij}|\tau_{i}\right)𝒩\left(\tau_{i},\rho_{i},\phi \right)p\left(\rho_{i}|\eta \right)p_{0}(\phi )𝒩(\eta ,\mu ,\Sigma )p_{0}(\mu )p_{0}(\Sigma )p_{0}(\beta )}{\int \prod_{i=1}^{I}\;\Pi_{j=1}^{J}\Pi_{t_{ij}=1}^{T_{ij}}p\left(r_{1:T_{ij}}|\theta_{ij},\;\beta ,\;c_{1:T_{ij}}\right)p\left(\theta_{ij}|\tau_{i}\right)𝒩\left(\tau_{i,},\rho_{i},\phi \right)p\left(\rho_{i}|\eta \right)p_{0}(\phi )𝒩(\eta ,\mu ,\Sigma )p_{0}(\mu )p_{0}(\Sigma )p_{0}(\beta )dX},$$

where the denominator is an integral across all possible values of X and is a constant for a given dataset and HBMc; $p_{0}(\mu )$, $p_{0}(\Sigma )$, $p_{0}(\phi )$, and $p_{0}(\beta )$ are the prior distributions of μ, Σ, ϕ, and β:

(16a) $$p_{0}(\mu )={𝒰}_{k}\left(\theta_{0k,min},\theta_{0k,max}\right),$$

(16b) $$p_{0}(\Omega )=𝒲\left(\Sigma_{HBMv}^{-1}/v,v\right),$$

(16c) $$p_{0}(\Sigma )=p_{0}\left(\Omega^{-1}\right),$$

(16d) $$p_{0}(\Lambda )=𝒲\left(\phi_{HBMv}^{-1}/\nu ,v\right),$$

(16e) $$p_{0}(\phi )=p_{0}\left(\Lambda^{-1}\right),$$

(16f) $$p_{0}(\beta )=𝒰(1,4),$$

where ${𝒰}_{k}\left(\theta_{0k,min},\theta_{0k,max}\right)$ denotes a uniform distribution between $\theta_{0k,min}$ and $\theta_{0k,max}$ at each of the K dimensions, with $\theta_{0k,min}$ and $\theta_{0k,max}$ defined in Table 2; $\Sigma_{HBMv}^{-1}$ was based on the covariance matrix of the estimated MCFF parameters, $\Sigma_{HBMv}$, across all subjects from the HBMv procedure; $\phi_{HBMv}^{-1}$ was based on the average covariance matrix $\phi_{HBMv}$ computed from the estimated MCFF parameters across all subjects from the HBMv procedure. Ω and Λ are K×K precision matrices with Wishart distributions 𝒲, with expected mean $\Sigma_{HBMv}^{-1}$ and $\phi_{HBMv}^{-1}$, and v=K+1 degrees of freedom.

The HBMc estimates the joint posterior distribution of MCFF parameters and hyperparameters as well as β across all tests and subjects. Unlike the HBMv, the HBMc generates a joint posterior distribution in which MCFF component parameter estimates mutually constrain each other across tests and subjects. This allows for more robust and interconnected estimates of MCFF parameters and hyperparameters.

### Computing the joint posterior distribution

We utilized R (R Core Team, 2003) function run.jags in JAGS (Plummer, 2003) to generate representative samples of $\theta_{ijk}\;(k=1,\;2,\ldots ,K)$ in three Markov Chain Monte Carlo (MCMC) chains for subject i, using the Bayesian Inference Procedure (BIP) through a random walk procedure (Kruschke, 2014). Each chain produced 5,000 kept samples (with a thinning ratioof 10) after a burn-in phase of 5,000 steps and 5,000 adaptation steps. Similarly, we computed 5,000 kept representative samples (with a thinning ratio of 10) of the joint posterior distribution of $\theta_{ijk}$ $\left(K\times 60\right.$ parameters), $\rho_{ik}$ $\left(K\times 60\right.$ parameters), $\sigma_{k}$ $\left(K\right.$ parameters), $\mu_{k}$ (K parameters), $\varepsilon_{k}$ (K parameters), and β (1 parameter) in three MCMC chains for HBMv after a burn-in phase of 5,000 steps and 5,000 adaptation steps. Additionally, we calculated 5,000 kept samples (with a thinning ratio of 10) of the joint posterior distribution of $\theta_{ij}$ (K×60 parameters), $\rho_{i}$ (K×60 parameters), Σ((K×K+K)/2 parameters). μ (K parameters). ϕ(((K×K+K)/2 parameters), and β (1 parameter) in three MCMC chains for HBMc after a burn-in phase of 500,000 steps and 5,000 adaptation steps. The number of adaptation steps was determined to ensure convergence, following Gelman and Rubin diagnostic rule (Gelman & Rubin, 1992). A model is considered “converged” when the between- and within-MCMC variance ratios for all parameters are less than 1.05. This ratio is calculated as the variance of samples across MCMC processed divided by the variance of samples within each MCMC process.

We applied the three modeling procedures to both pre-training and training data utilizing the most saturated model (K=14). For the HBMc, two reduced models (K=2,6) were also fit. Additionally, the most saturated HMBc was fit to augmented data, which included full datasets of 59 subjects. Predicted learning curves were generated for a sample subject i=15 under various scenarios, including no data, one session of data, or two sessions of data; and these predictions were compared with the actual learning curve of this subject.

### Statistical analysis

We initially estimated the $\theta_{ij}$ for all subjects using the BIP, HBMv, and HBMc, regardless of training accuracy and feedback conditions. In this study, j was set to 1 since each subject underwent testing only once. Subsequently, we computed the estimated trial-by-trialthreshold learning curve, $\xi_{t_{i1}}$, from the estimated $\theta_{i1}$ for each subject, and unblinded the data to compute group-level statistics of the estimated learning curve for each group g.

#### Goodness of fit

We assessed and compared goodness of fit among the three methods using the Bayesian predictive information criterion (BPIC) (Ando, 2007, 2011), which quantifies the likelihood of the data based on the joint posterior distribution of MCFF parameters while also penalizing for model complexity.

#### Mean and Standard Deviation of $\xi_{t_{i1}}$

The posterior distribution of $\xi_{t_{i1}}$ was constructed by computing the learning curve (Figure 1) from samples of the posterior distribution of $\theta_{i1}$. To measure variability or uncertainty at the test level for the BIP, HBMv, and HBMc methods, we utilized the half width of the 68.2% credible interval (HWCI) of the posterior distribution of $\xi_{t_{i1}}$ (Clayton & Hills, 1993; Edwards et al., 1963).

#### Group-level statistics

The posterior distribution $p\left(\theta_{g}\right)$ of the MFCC parameters of the learning curve of group g, $\theta_{g}$, was constructed by (1) averaging each sample $\theta_{i1}$ from the joint distribution at the test level across all subject i in the group, and (2) repeating (1) 15,000 times. One-way MANOVA was conducted on the mean ${\overset{‾}{\theta }}_{g}$ of $p\left(\theta_{g}\right)$ with R function ***manova***. Linear discriminant analysis (LDA) was conducted on the $p\left(\theta_{g}\right)$ with R function ***lda*** for post hoc comparisons. In each LDA, 90% and 10% random samples from $p\left(\theta_{g}\right)$ were used as training and test data, respectively. The LDA was repeated 100 times.

## RESULTS

### Goodness of fit

The Bayesian Predictive Information Criterion (BPIC) values for the most saturated model (K=14) were 125144, 125119 and 125047 for the BIP, HBMv, and HBMc, respectively. These values indicated that the HBMc provided the best fit to the data among the three models.

For the HBMc, when constraining $\delta_{s}$ and $d_{s}$ to be the same across sessions (K=6), the BPIC value increased to 125056. Retaining only general learning (K=2) further increased the BPIC to 126286. As a result, it was concluded that the HBMc with $\delta_{s}$ and $d_{s}$ varying across sessions (K=14) produced the best fit to the data.

### Comparing BIP, HBMv and HBMc learning curves

**Figure 3.**
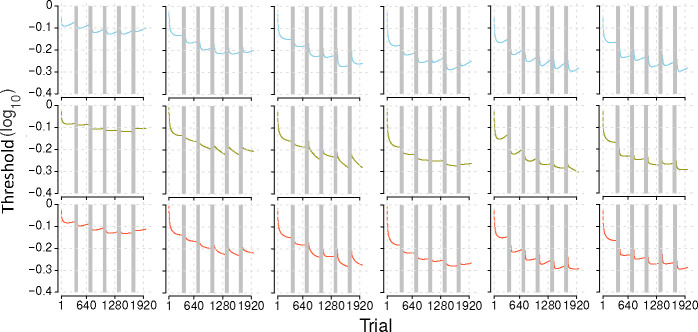
Average trial-by-trial learning curves across all subjects in groups 1 to 6 (columns 1 to 6), with mean (lines) and standard error (shaded areas) from the three methods: BIP (blue), HBMv (olive), HBMc (orange).

Figure 3 illustrates the mean and standard error of the average trial-by-trial learning curves for each of the six groups from the most saturated MCFF generated with the BIP, HBMv, and HBMc. The standard errors in Groups 3 and 4 are larger because they only contained six subjects each, while the other groups had 12 subjects each. Table 3 presents the average 68.2% half width credible interval (HWCI; in ${log}_{10}$ threshold units) of the learning curves in the six groups. The HBMc generated the most precise estimates of the learning curves, with the smallest average 68.2% HWCI in all groups. Based on these and the BPIC results, we will focus on the HBMc with K=14 in subsequent analyses.

### Posterior distributions from the HBMc

**Figure 4.**
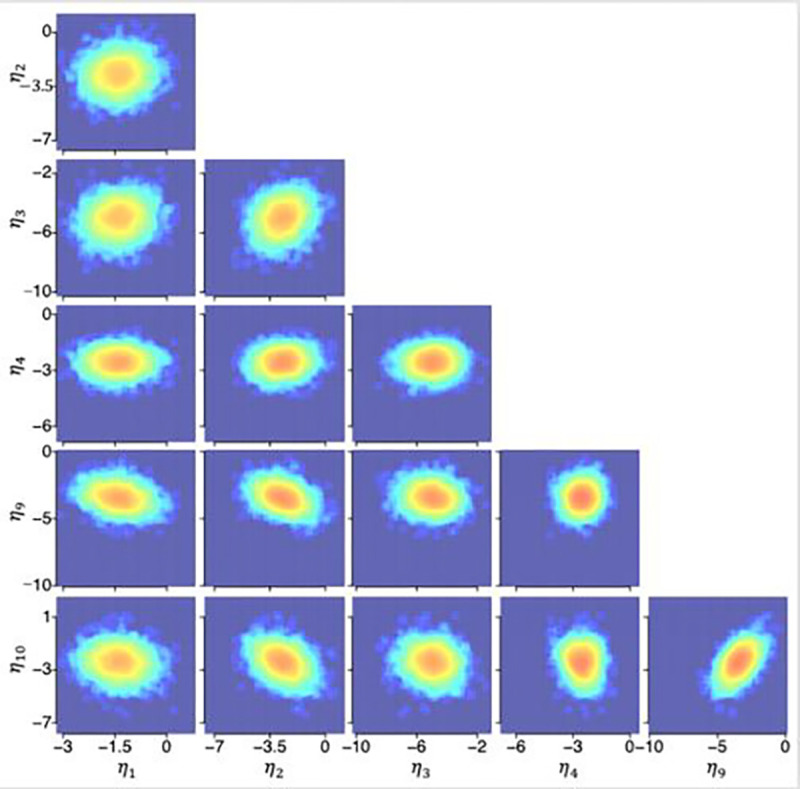
Posterior distributions of hyperparameters η at the population level.

**Figure 5.**
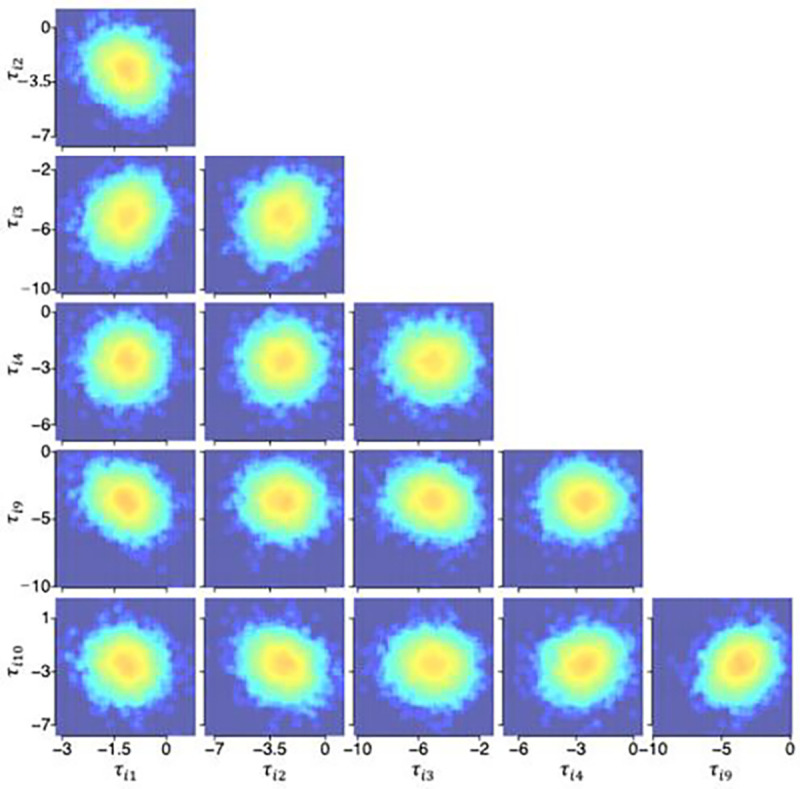
Posterior distributions of hyperparameters $\tau_{i}(i=15)$ at the subject level.

**Figure 6.**
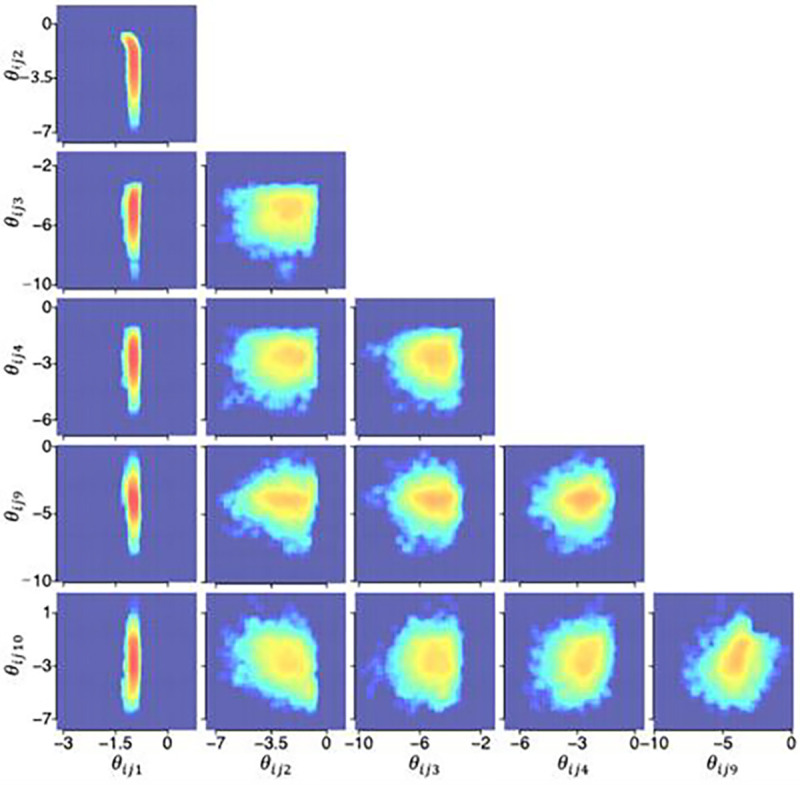
Posterior distributions of hyperparameters $\theta_{ij}(i=15,j=1)$ at the test level.

Figures 4, 5, and 6 illustrate the posterior distributions of MCFF hyperparameters and parameters at the population, subject, and test levels obtained from the HBMc with K=14 as two-dimensional projections between pairs of six representative dimensions (k=1,2,3,4,9,10) of the MCFF parameters in the HBMc, with only one of the between-session forgetting parameters and one of the asymptotic rapid relearning levels. The HBMc, with its incorporation of covariance hyperparameters, recovered the relationships between MCFF parameters among different subjects and tests. Correlations ranged from −0.47 to 0.64, −0.31 to 0.50, −0.74 to 0.66, at the population, subject, test levels, respectively (Tables 4, 5, and 6). In addition, the mean and 68.2% HWCI of the β posterior distribution were 2.00 and 0.041, respectively.

### Group-level statistics

**Figure 7.**
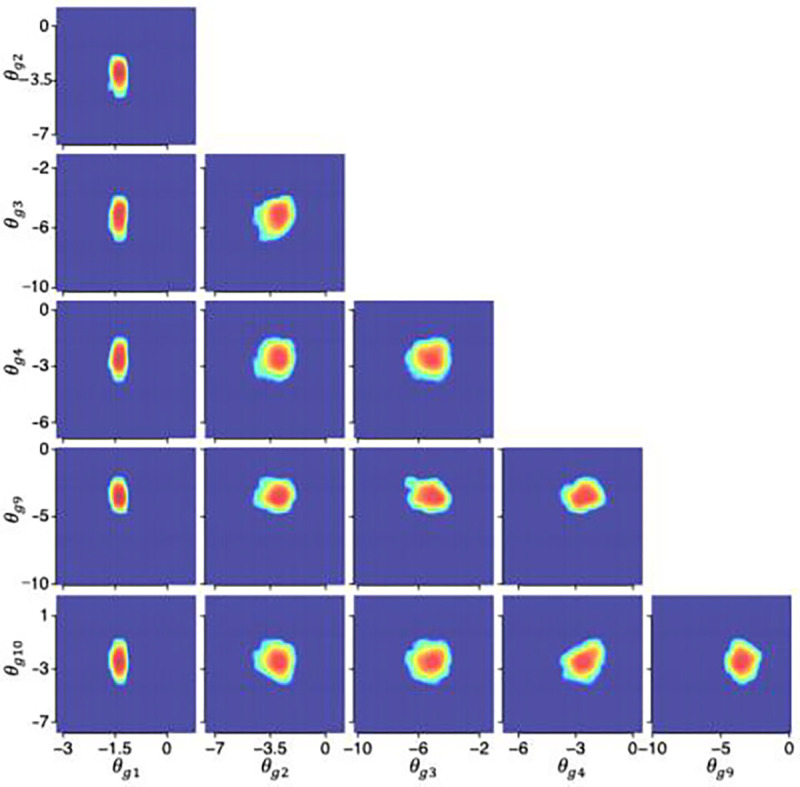
Posterior distributions of $\theta_{g}(g=2)$.

In order to conduct group-level analysis, we constructed the joint distributions of the MCFF parameters $p\left(\theta_{g}\right)$ at the group level. Figure 7 illustrates $p\left(\theta_{g}\right)$ of group 2. Table 7 shows the correlations of $\theta_{g}$ components in the group. Again, the HBMc recovered the relationships between MCFF parameters at the group level. For groups 1 to 6, correlations of $\theta_{g}$ components ranged from −0.44 to 0.52, −0.31 to 0.50, −0.35 to 0.46, −0.37 to 0.55, −0.38 to 0.55, and −0.33 to 0.50, respectively.

One-way MANOVA on ${\overset{‾}{\theta }}_{g}$ across all six groups showed a significant main effect of group [F(70,225)=1.54,p=0.0096]. As shown in Table 8, LDA on the $\theta_{g}$ distributions revealed that the group in low training accuracy without feedback (Group 1) can be accurately separated from the other five groups (average accuracy: 98±2%), whereas the other groups (Groups 2 to 6) were less separated from each other (average accuracy: 87±7%). When we performed a one-way MANOVA on ${\overset{‾}{\theta }}_{g}$ from Groups 2 to 6, the main effect of group was not significant [F(56,132)=,p=0.15].

We also computed the mean and 68.2%HWCI of the marginal distributions of the general learning rate in each group. Although there was significant general learning in all groups, Group 1 exhibited a significantly lower learning rate (−0.026±0.006) compared to the other five groups (−0.041±0.005, −0.043±0.010, −0.064±0.009, −0.046±0.006, and −0.044±0.007).

### Predicting the learning curve

**Figure 8.**
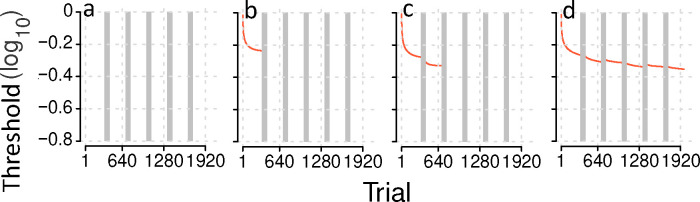
Mean (lines) and 68.2%HWCI (shaded areas) of the observed (orange) and predicted (yellow) trial-by-trial learning curves from the HBMc with (a) no, (b) one session, and (c) two sessions of data for subject i=15. (d) The observed learning curve of subject i=15 with all six sessions of data.

Figure 8 illustrates the predicted learning curves for subject i=15 with no data, one session, and two sessions, along with the actual observed learning curve across all six sessions. In this figure, we colored the segments of the estimated learning curves from observed data in orange and those from predicted curves in yellow. The correlations between the predicted and observed learning curves (with all six sessions of data) were 0.841,0.944, and 0.958 when there was no data, one session, and two sessions, respectively. The average 68.2% HWCI of the predicted learning curves was 0.278, 0.077, and 0.055log_10_ units for scenarios with no data, one session, and two sessions, corresponding to 1252%, 331%, and 238% increase of 68.2% HWCI compared to the actual observed learning curve with all six sessions of data. Both the accuracy and the reliability of the predicted learning curves increased with the amount of available data. Remarkably, the predicted learning curves from one and two sessions of data closely matched that of the observed learning curve for this subject.

## DISCUSSION

We introduced the multi-component functional form (MCFF) from the non-parametric HBMc analysis (Zhao et al. submitted) along with the contrast threshold psychometric function as the generative model of trial-by-trial performance in perceptual learning. We developed three parametric inference procedures to estimate the parameters of the MCFF and, therefore, the trial-by-trial learning curve from two datasets that investigated the interaction between feedback and training accuracy in Gabor orientation identification.

The HBMc incorporated covariance hyperparameters at the population and subject levels, capturing the relationship between and within subjects, resulting in the best fits to the trial-by-trial data and precise estimates of the learning curves. Among the HBMc solutions with different numbers of MCFF components, the HBMc with the most saturated version (K=14) generated the best fit to the trial-by-trial data, aligning with findings in our non-parametric HBMc analysis (Zhao et al. submitted) and extending them to individual subject levels.

MANOVA and LDA analyses of the joint distributions at the group level found that the low-training accuracy without feedback group (Group 1) was significantly different from the other groups that received training either at a high accuracy and/or with feedback (Groups 2 to 6). We also found significant general learning in all groups, although Group 1 exhibited a significantly lower learning rate compared to the other five groups. These findings align with the results from the original studies (Liu et al., 2010, 2012) as well as the non-parametric HBMc analysis (Zhao et al. submitted), although both the trial-by-trial analysis with parametric HBMc and the 20-trial-block analysis with non-parametric HBMc are shown to be more sensitive to detect significant learning in Group 1, which the traditional adaptive staircase method failed to detect.

The HBMc provided population-level and individual-level posterior distributions of model parameters. This feature facilitated statistical inferences and predictions of the trial-by-trial learning curve. Even with minimal data, the predictions demonstrated a notable correlation with observed data, highlighting the HBMc’s potential to enhance test efficiency.

Accurate predictions of the learning curve could hold significant implications, especially in fields requiring perceptual expertise. This includes occupations in which selecting individuals with the aptitude for efficient perceptual learning is crucial. Moreover, it opens avenues for optimizing training strategies and protocols, particularly in clinical applications, rehabilitation settings, and domains demanding perceptual expertise.

Training for perceptual expertise is often resource-intensive, both in terms of cost and time (Dosher & Lu, 2020). Therefore, the ability to selectively identify individuals with a higher likelihood of acquiring the necessary perceptual expertise efficiently has the potential to increase the overall success rate of training programs.

Although our primary focus has been on the estimation of the trial-by-trial learning curve, the methods developed here can also be used to study specificity and transfer in perceptual learning. Studies on specificity or transfer in perceptual learning have mostly relied on the estimates of initial transfer, usually quantified as performance during the first assessment after a task switch. Some studies, though fewer in number, have examined learning rates following a task switch and have reported instances of accelerated learning or ‘learning to learn’ (Bejjanki et al., 2014; Kattner, Cochrane, Cox, et al., 2017; Z. L. Liu & Weinshall, 2000; R.-Y. Zhang et al., 2021). However, the granularity of analysis in these studies has often been coarse, potentially masking multiple underlying component processes that may be affected by prior learning. The parametric methods developed in this study could be used to recover trial-by-trial learning curves and the time course of transfer for each subject.

While some researchers have advocated for modeling perceptual learning as a continuous function of time-on-task, applying parametric functional forms such as exponential or power curves (Cochrane, et al, submitted), our previous study (Zhao, et al., submitted) revealed the intricacies of the learning process. This process extends beyond general learning, encompassing phenomena like between-session forgetting and within-session rapid relearning and adaptation.

In our prior work, we exclusively applied the non-parametric methods to analyze the contrast threshold learning curve in an orientation identification task. Moving forward, our strategy involves applying the non-parametric approach to diverse perceptual learning tasks. This broader application aims to identity the specific component processes at play in different tasks. Subsequently, we plan to leverage the insights gained to apply the parametric methods presented in this study, enabling the estimation of trial-by-trial learning curves across various perceptual learning domains. Such finer-grained learning curves may be more sensitive to dynamic training and transfer effects, revealing a more nuanced and complete picture of perceptual learning.

Additionally, the Multi-Component Functional Form (MCFF), besides enabling the estimation of trial-by-trial learning curve, may be valuable for enhancing adaptive assessment in perceptual learning testing procedures and monitoring the time course of perceptual sensitivity change. In our previous work, we introduced the adaptive qCD method to assess perceptual sensitivity changes (e.g., dark adaptation, perceptual learning) with an exponential functional form (Zhao et al., 2019). The qCD showcased its efficacy in estimating trial-by-trial learning curves with high accuracy and precision compared to traditional staircase procedures (Zhang et al., 2019b). With the refined functional form provided by the MCFF, to the qCD method can be further enhanced to achieve a more accurate estimation of the time course of learning.

Although we have developed the HBMc with MCFF within the context of perceptual learning, the parametric framework can be harnessed to quantify learning curves in different learning domains. It can also be employed to improve estimates of human performance parameters, such as $d^{\prime }$, response time, and threshold, across multiple time points in learning or longitudinal studies, or across diverse experimental conditions, such as different spatial frequencies in contrast sensitivity function (CSF) tests or varying temporal frequencies in assessments of temporal modulation functions.

In summary, the MCFF implemented in the parametric HBMc serves as a powerful tool for estimating trial-by-trial learning curves, revealing detailed time courses of processes in perceptual learning. Its versatility provides an effective framework for generating accurate and precise estimates as well as predictions of dynamics changes in human performance across experiments with hierarchical designs.

## Figures and Tables

**Table 1:** Correspondence between $\theta_{ijk}$ and original MCFF parameters.

	*K* = 14	*K* = 6	*K* = 2
${log}_{10}(\gamma )$	$\theta_{ij1}$	$\theta_{ij1}$	$\theta_{ij1}$
${log}_{10}(-b)$	$\theta_{ij2}$	$\theta_{ij2}$	$\theta_{ij2}$
${log}_{10}\left(\varphi_{s}\right)$	$\theta_{ij3}$	$\theta_{ij3}$	
${log}_{10}\left(\delta_{s}\right)$	$\theta_{ij4},\theta_{ij5},\theta_{ij6},\theta_{ij7},\theta_{ij8}$	$\theta_{ij4}$	
${log}_{10}\left(\tau_{s}\right)$	$\theta_{ij9}$	$\theta_{ij5}$	
${log}_{10}\left(d_{s}\right)$	$\theta_{ij10},\theta_{ij11},\theta_{ij12},\theta_{ij13},\theta_{ij14}$	$\theta_{ij6}$	

**Table 2.** Lower and upper bounds of priors of MCFF parameters.

*k*				
*K* = 14	*K* = 6	*K* = 2	$\theta_{0k,min}$	$\theta_{0k,max}$
1	1	1	−3	−0.5
2	2	2	−3	−0.5
3	3		−6	−3
4,5,6,7,8	4		−3	−1
9	5		−4	−2
10,11,12,13,14	6		−3	−0.6

**Table 3.** The average 68.2% half width credible interval (HWCI; in log_10_ threshold units) of the learning curves in the six groups.

Group	1	2	3	4	5	6

BIP	0.034	0.037	0.031	0.028	0.025	0.025
HBMv	0.029	0.031	0.031	0.023	0.023	0.024
HBMc	0.029	0.030	0.030	0.022	0.022	0.023

**Table 4.** Correlations of η components.

	$\eta_{1}$	$\eta_{2}$	$\eta_{3}$	$\eta_{4}$	$\eta_{5}$	$\eta_{6}$	$\eta_{7}$	$\eta_{8}$	$\eta_{9}$	$\eta_{10}$	$\eta_{11}$	$\eta_{12}$	$\eta_{13}$

$\eta_{2}$	0.12												
$\eta_{3}$	0.12	0.24											
$\eta_{4}$	−0.03	0.08	0.06										
$\eta_{5}$	0.09	0.32	0.17	−0.07									
$\eta_{6}$	0.07	0.19	0.06	−0.13	0.16								
$\eta_{7}$	0.09	0.33	−0.07	0.04	0.01	0.02							
$\eta_{8}$	−0.06	0.08	−0.03	−0.07	−0.10	0.05	0.01						
$\eta_{9}$	−0.29	−0.40	−0.16	0.06	−0.23	−0.08	−0.18	−0.25					
$\eta_{10}$	−0.12	−0.37	−0.10	−0.12	−0.29	−0.03	−0.07	−0.19	0.46				
$\eta_{11}$	−0.19	−0.43	−0.13	0.06	−0.38	−0.21	−0.24	−0.07	0.53	0.61			
$\eta_{12}$	−0.31	−0.45	−0.14	0.06	−0.22	−0.33	−0.22	−0.18	0.51	0.47	0.51		
$\eta_{13}$	−0.26	−0.45	−0.10	−0.01	−0.22	−0.13	−0.47	−0.17	0.51	0.47	0.61	0.64	
$\eta_{14}$	−0.19	−0.43	−0.12	0.08	−0.16	−0.20	−0.24	−0.35	0.47	0.55	0.64	0.53	0.63

**Table 5.** Correlations of $\tau_{i}$ components, averaged across all subjects.

	$\tau_{i1}$	$\tau_{i2}$	$\tau_{i3}$	$\tau_{i4}$	$\tau_{i5}$	$\tau_{i6}$	$\tau_{i7}$	$\tau_{i8}$	$\tau_{i9}$	$\tau_{i10}$	$\tau_{i11}$	$\tau_{i12}$	$\tau_{i13}$

$\tau_{i2}$	−0.16												
$\tau_{i3}$	0.15	0.16											
$\tau_{i4}$	−0.02	0.04	0.04										
$\tau_{i5}$	0.07	0.13	0.12	0.00									
$\tau_{i6}$	0.04	0.06	0.05	−0.03	0.09								
$\tau_{i7}$	0.07	0.10	0.01	0.01	0.01	0.04							
$\tau_{i8}$	−0.01	−0.02	0.01	0.00	−0.03	0.03	0.04						
$\tau_{i9}$	−0.27	−0.16	−0.21	0.05	−0.12	−0.03	−0.08	−0.08					
$\tau_{i10}$	−0.09	−0.16	−0.02	0.16	−0.09	−0.03	−0.02	−0.06	0.15				
$\tau_{i11}$	−0.11	−0.16	−0.01	0.07	−0.04	−0.08	−0.06	0.00	0.12	0.39			
$\tau_{i12}$	−0.20	−0.16	−0.05	0.04	−0.07	0.01	−0.06	−0.07	0.15	0.26	0.25		
$\tau_{i13}$	−0.14	−0.14	0.00	−0.01	−0.06	−0.02	−0.03	−0.05	0.07	0.23	0.36	0.36	
$\tau_{i14}$	−0.13	−0.13	−0.01	0.07	−0.02	−0.06	−0.07	0.01	0.04	0.33	0.42	0.28	0.38

**Table 6.** Correlations of $\theta_{ij}$ components, averaged across all subjects.

	$\theta_{ij1}$	$\theta_{ij2}$	$\theta_{ij3}$	$\theta_{ij4}$	$\theta_{ij5}$	$\theta_{ij6}$	$\theta_{ij7}$	$\theta_{ij8}$	$\theta_{ij9}$	$\theta_{ij10}$	$\theta_{ij11}$	$\theta_{ij12}$	$\theta_{ij13}$

$\theta_{ij2}$	−0.27												
$\theta_{ij3}$	0.23	0.13											
$\theta_{ij4}$	0.03	0.05	0.05										
$\theta_{ij5}$	0.06	0.11	0.09	0.02									
$\theta_{ij6}$	0.03	0.04	0.04	−0.02	0.07								
$\theta_{ij7}$	0.03	0.07	−0.01	0.02	0.00	0.03							
$\theta_{ij8}$	0.05	−0.03	0.01	0.01	−0.02	0.03	0.04						
$\theta_{ij9}$	−0.28	−0.05	−0.15	0.00	−0.07	0.00	−0.01	−0.05					
$\theta_{ij10}$	−0.08	−0.11	0.05	0.19	−0.06	−0.01	0.00	−0.03	0.02				
$\theta_{ij11}$	−0.10	−0.12	0.05	0.05	0.02	−0.06	−0.04	0.00	−0.02	0.38			
$\theta_{ij12}$	−0.14	−0.12	0.01	0.01	−0.04	0.05	−0.04	−0.06	0.00	0.25	0.26		
$\theta_{ij13}$	−0.12	−0.11	0.04	−0.02	−0.05	−0.01	0.02	−0.05	−0.05	0.24	0.37	0.39	
$\theta_{ij14}$	−0.11	−0.11	0.04	0.04	−0.01	−0.04	−0.05	0.04	−0.07	0.33	0.44	0.30	0.40

**Table 7.** Correlations of $\theta_{g}$ components (g=2).

	$\theta_{g1}$	$\theta_{g2}$	$\theta_{g3}$	$\theta_{g4}$	$\theta_{g5}$	$\theta_{g6}$	$\theta_{g7}$	$\theta_{g8}$	$\theta_{g9}$	$\theta_{g10}$	$\theta_{g11}$	$\theta_{g12}$	$\theta_{g13}$

$\theta_{g2}$	−0.09												
$\theta_{g3}$	0.24	0.21											
$\theta_{g4}$	0.11	0.04	0.05										
$\theta_{g5}$	0.02	0.17	0.08	0.06									
$\theta_{g6}$	−0.05	−0.01	0.03	−0.06	0.04								
$\theta_{g7}$	0.07	0.04	0.00	0.00	−0.03	−0.03							
$\theta_{g8}$	−0.02	−0.03	−0.01	−0.05	0.01	0.00	−0.02						
$\theta_{g9}$	−0.23	−0.05	−0.28	−0.07	−0.02	0.00	0.08	−0.09					
$\theta_{g10}$	−0.07	−0.08	0.17	0.30	0.07	0.03	−0.04	−0.05	−0.15				
$\theta_{g11}$	−0.08	−0.08	0.10	0.06	0.12	−0.01	−0.04	0.07	−0.22	0.43			
$\theta_{g12}$	−0.14	−0.09	0.08	−0.06	0.02	0.06	−0.09	−0.06	−0.20	0.28	0.34		
$\theta_{g13}$	−0.06	−0.14	0.12	−0.04	−0.01	0.01	−0.04	0.02	−0.31	0.32	0.47	0.50	
$\theta_{g14}$	−0.06	−0.12	0.11	0.04	0.02	−0.01	−0.06	0.12	−0.28	0.35	0.47	0.33	0.48

**Table 8.** Average accuracy (% correct) and standard deviation of linear discriminant analyses.

Group	1	2	3	4	5

2	95 (0.4)				
3	98 (0.3)	89 (0.6)			
4	100 (0.0)	96 (0.4)	92 (0.6)		
5	98 (0.2)	83 (0.7)	90 (0.6)	92 (0.5)	
6	99 (0.2)	79 (0.9)	84 (0.7)	94 (0.5)	73 (1.0)

## Data Availability

The data that support the findings of this study are available from the corresponding author upon request.
